# Complications of extraperitoneal robot-assisted radical prostatectomy in high-risk prostate cancer: A single high-volume center experience

**DOI:** 10.3389/fsurg.2023.1157528

**Published:** 2023-03-30

**Authors:** Alessio Paladini, Giovanni Cochetti, Graziano Felici, Miriam Russo, Eleonora Saqer, Luigi Cari, Stefano Bordini, Ettore Mearini

**Affiliations:** ^1^ Urology Clinic, Department of Medicine and Surgery, University of Perugia, Perugia, Italy; ^2^ Section of Pharmacology, Department of Medicine and Surgery, University of Perugia, Perugia, Italy

**Keywords:** prostate cancer, robot-assisted, radical prostatectomy, extraperitoneal, complications, lymph node dissection

## Abstract

**Introduction:**

The role of robot-assisted radical prostatectomy (RARP) in high-risk prostate cancer (PCa) has been debated over the years, but it appears safe and effective in selected patients. While the outcomes of transperitoneal RARP for high-risk PCa have been already widely investigated, data on the extraperitoneal approach are scarcely available. The primary aim of this study is to evaluate intra- and postoperative complications in a series of patients with high-risk PCa treated by extraperitoneal RARP (eRARP) and pelvic lymph node dissection. The secondary aim is to report oncological and functional outcomes.

**Methods:**

Data of patients who underwent eRARP for high-risk PCa were prospectively collected from January 2013 to September 2021. Intraoperative and postoperative complications were recorded, as also perioperative, functional, and oncological outcomes. Intraoperative and postoperative complications were classified by employing Intraoperative Adverse Incident Classification by the European Association of Urology and the Clavien–Dindo classification, respectively. Univariate and multivariate analyses were performed to evaluate a potential association between clinical and pathological features and the risk of complications.

**Results:**

A total of 108 patients were included. The mean operative time and estimated blood loss were 183.5 ± 44 min and 115.2 ± 72.4 mL, respectively. Only two intraoperative complications were recorded, both grade 3. Early complications were recorded in 15 patients, of which 14 were of minor grade, and 1 was grade IIIa. Late complications were diagnosed in four patients, all of grade III. Body mass index (BMI) > 30 kg/m^2^, Prostate-Specific Antigen (PSA) > 20 ng/mL, PSA density >0.15 ng/mL^2^, and pN1 significantly correlated with a higher rate of overall postoperative complications. Moreover, BMI >30 kg/m^2^, PSA >20 ng/mL, and pN1 significantly correlated with a higher rate of early complications, while PSA >20 ng/mL, prostate volume <30 mL, and pT3 were significantly associated with a higher risk of late complications. In multivariate regression analysis, PSA >20 ng/mL significantly correlated with overall postoperative complications, while PSA > 20 and pN1 correlated with early complications. Urinary continence and sexual potency were restored in 49.1%, 66.7%, and 79.6% of patients and in 19.1%, 29.9%, and 36.2% of patients at 3, 6, and 12 months, respectively.

**Conclusions:**

eRARP with pelvic lymph node dissection in patients with high-risk PCa is a feasible and safe technique, resulting in only a few intra- and postoperative complications, mostly of low grade.

## Introduction

Prostate cancer (PCa) affects over one million men a year and is the most common non-cutaneous malignancy in males. For localized PCa, minimally invasive radical prostatectomy (RP) is the gold standard surgical therapy in addition to open radical prostatectomy (1). Unfortunately, almost 25% of patients are diagnosed with high-risk disease and these patients are at a higher risk of biochemical recurrence, metastatic progression, and cancer-specific mortality. Therefore, the best therapeutic strategies for localized and locally advanced high-risk PCa are still being debated (1).

Indeed, RP shows good oncological outcomes and survival benefits in patients with high-risk PCa, even though it is unclear whether RP is superior to radiation therapy combined with androgen deprivation therapy (2, 3). There are several studies in the literature, mostly retrospective, that compare the two treatments, however with discordant results (1).

According to the European Association of Urology (EAU), RP may be proposed as a first-line therapy in high-risk PCa as part of a multimodality strategy (4).

Furthermore, EAU guidelines recommend that extended pelvic lymph node dissection (ePLND) to be performed according to validated nomograms; patients with lymph node involvement risk constitute >5% according to Briganti's or Memorial Sloan Kettering nomogram, or those with risk >7% according to Gandaglia's nomogram shall undergo ePLND. Although ePLND remains the most accurate method for staging PCa confined to the pelvis, its therapeutic benefit is still unclear (5–7). ePLND provides better pathological staging than standard PLND, facilitating subsequent multimodality treatments, while no difference in oncological outcomes has been demonstrated (4, 8, 9).

Robot-assisted radical prostatectomy (RARP) with the transperitoneal approach (tRARP) is the most common technique because it allows a wide surgical field facilitating the execution of PLND with an extended template. Extraperitoneal RARP (eRARP) is an alternate technique with similar outcomes. Its advantages consist in reducing the Trendelenburg position, thanks to Retzius space gas pressure, which not only pushes up the peritoneum but also functions as a natural retractor preventing bowel displacement into the surgical field (10–12). A recent meta-analysis, comparing the two techniques, showed a shorter operative time and length of stay, lower bleeding, and rate of minor complications (13). Horovitz et al., in a prospective study comparing eRARP and tRARP, found a lower rate of ileus, overall complications, and a shorter length of stay (8). While outcomes, including the complication rate, of tRARP for high-risk PCa have been already widely investigated, data on eRARP are scarcely available.

The main purpose of this study was to evaluate intra- and postoperative complications in a series of patients affected by high-risk PCa treated by eRARP and PLND. The secondary aim was to report oncological and functional outcomes.

## Materials and methods

We prospectively collected data of patients who underwent eRARP at a high-volume center from January 2013 to September 2021. All surgeries were performed by a single experienced surgeon. To each patient was offered all treatments recommended by the EAU guidelines in accordance with the risk of the disease.

The inclusion criteria were the following: (1) patients with preoperative localized high-risk PCa; (2) at least 1-year of follow-up. High-risk localized PCa was defined using the EAU risk group according to EAU-ESTRO-SIOG guidelines (4).

The exclusion criteria were the following: (1) life expectancy <10 years; (2) administration of neoadjuvant androgen deprivation therapy; (3) previous history of pelvic radiation therapy or major pelvic surgery; (4) previous history of urinary incontinence or urethral stricture.

eRARP was performed according to the PERUSIA technique (10, 11). This technique is particularly useful in low-risk PCa and the key points are the following: (1) the perpendicular approach to the medial aspect of seminal vesicles and their mobilization from the medial to the lateral side to minimize the manipulation of the neurovascular bundles, in case of their preservation; (2) anterograde dissection; (3) preservation of the anterior peri-prostatic tissue; 4) preservation of the deep venous complex. In our case series, which included only patients with high-risk PCa, the veil of Aphrodite, deep venous complex, as well as neurovascular bundles were not preserved.

PLND was performed in all patients and the extended template was applied when the risk of lymph node involvement was >5% according to the memorial Sloan–Kettering nomogram, and since 2019 when the risk was >7% according to Gandaglia's nomogram (5, 7).

Preoperative, intraoperative, and postoperative data were prospectively collected. The following preoperative demographic information was evaluated: age, body mass index (BMI), Charlson comorbidity index, American Society of Anaesthesiologists score, prostate volume, prostate-specific antigen (PSA) level, PSA density, biopsy Gleason score, biopsy International Society of Urological Pathology (ISUP) grade group, and cTNM. Urinary continence was evaluated through direct interview according to question number 3 of the Expanded Prostate Cancer Index Composite (EPIC) questionnaire (14). Patients using no pads were considered continent. Urinary symptoms and sexual function were assessed using the International Prostate Symptom Score (IPSS) and the 5-item International Index of Erectile Function (IIEF-5), respectively (15, 16). Patients were defined potent when the IIEF-5 score was ≥17.

The evaluated perioperative outcomes were operative time, estimated blood loss, rate of blood transfusions, complication rate, catheterization time, and length of stay. Intraoperative and postoperative complications were recorded and evaluated using the Intraoperative Adverse Incident Classification by the European Association of Urology (EAUiaiC) and the Clavien–Dindo classification, respectively (17, 18). All patients underwent predischarge pelvic ultrasound to evaluate fluid pelvic collections.

Complications were assessed and divided as early (≤30 days) and late (>30 days).

All RARP histology reports were collected. PSA was detected within 6–8 weeks from surgery, every 6 months until 3 years, and yearly thereafter. IIEF-5, IPSS, and 3-item EPIC were assessed at 3, 6, and 12 months, respectively. Sexual function was evaluated in yet preoperative potency patients as a recovery of IIEF-5 ≥ 17.

The study population was stratified according to age (<70 vs. ≥70 years), BMI (<25 vs. 25–30 vs. >30 kg/m^2^), PSA (<10 vs. 10–20 vs. >20 ng/mL), PSA density (≤0.15 vs. >0.15 ng/mL^2^), prostate volume (<30 vs. 30–60 vs. >60 mL), ISUP (1 vs. 2–3 vs. 4–5), pT (pT2 vs. pT3), and pN (pN0 vs. pN1), and univariate and multivariate logistic regression analyses were performed to determine a potential association with the risk of complications.

The study was conducted in accordance with the Declaration of Helsinki. Ethical review and approval were waived for this study because of the use of the gold standard treatment for the disease according to the European Association of Urology Guidelines.

### Statistical analysis

Individual variables were stratified and the percentage of patients with postoperative complications in each subgroup was calculated (complication rate); the contingency test (Chi-square test) was used to evaluate the statistical significance of the complication rate (overall complications, early complications, and late complications). The multiple logistic regression test was used for the combined analysis of variables; the odds ratio values (95% CI) and *p* values were reported.

Statistical significance was set at *p* < 0.05. Statistical analysis was conducted using SPSS® Statistics Software.

## Results

Overall, 1,188 patients with PCa were treated by eRARP in our center. Data on 177 patients with high-risk localized and locally advanced PCa were collected. Of these, 69 patients were excluded because of early dropout from the study on their own request. Finally, 108 patients were included in the study.

The mean age and PSA were 66.8 ± 5.2 years and 9.9 ± 6.8 ng/mL, respectively. Twenty-seven patients were classified as cT1c, seventy-eight were cT2, and three cT3. Other demographic and clinical–pathological characteristics are reported in Table 1.

**Table 1:** Demographic and preoperative clinicopathological characteristics.

	Mean ± SD
Age (year)	66.8 ± 5.2
BMI (kg/m^2^)	26.5 ± 2.4
Charlson comorbidity index	2.4 ± 0.9
PSA (ng/mL)	9.9 ± 6.8
PSA density (ng/mL^2^)	0.2 ± 0.2
Prostate volume (mL)	49.5 ± 26.5
ISUP grade (*n*)	
I	17
II	24
III	21
IV	32
V	14
Clinical T-stage (*n*)	
cT1c	27
cT2a	21
cT2b	24
cT2c	33
cT3	3
IPSS	10.4 ± 7.1
Continence (%)	100%
IIEF-5	14.5 ± 7.3
Potency (%)	43.5%

BMI, body mass index; IPSS, International Prostate Symptom Score; IIEF-5, International Index of Erectile Function; PSA, prostate-specific antigen.

The mean operative time was 183.5 ± 44.0 min, and estimated blood loss was 115.2 ± 72.4 mL. Overall, two intraoperative complications were recorded, both of grade 3, according to EAUiaiC: one was a bleeding from the inferior epigastric vein during trocar insertions treated intraoperatively with coagulation and clip; this was the only case of a patient needing blood transfusion. The other intraoperative complication was a pneumothorax that needed postoperative drainage.

In the final specimen, 14 patients were classified as ISUP 1, 22 ISUP 2, 29 ISUP 3, 27 ISUP 4, and 16 ISUP 5. The pathological stage revealed 53 pT2, 37 pT3a, and 18 pT3b. Positive surgical margins were recorded in 29.6% of patients. The mean number of lymph node removed was 12.2 ± 5.3 and nine patients were classified as pN1. The median time of catheterization and pelvic drain stay were 7 days (range 6–14). The median length of stay was 7 days (range 3–10). The mean follow-up was 50.6 ± 36.7 months.

The overall rate of complications was 17.6%. Early complications were recorded in 15 patients (13.9%), with 14 (13%) of minor grade and 1 (0.9%) of grade IIIa. Five patients suffered anastomosis leakage, which was treated with catheter substitution and maintained for 10 days; nine cases of patients with asymptomatic lymphoceles were detected by using predischarge pelvic ultrasound, and these patients did not need any other treatment; one patient with symptomatic lymphocele was treated by drain placement under ultrasound guidance. Late complications were diagnosed in four patients (3.7%), all of grade III. One symptomatic lymphocele was treated with drain placement under CT-scan guidance (grade IIIa); two bladder neck contractions and one Hem-o-Lok anastomosis migration were treated by endoscopic surgery (grade IIIb).

**Figure 1:**
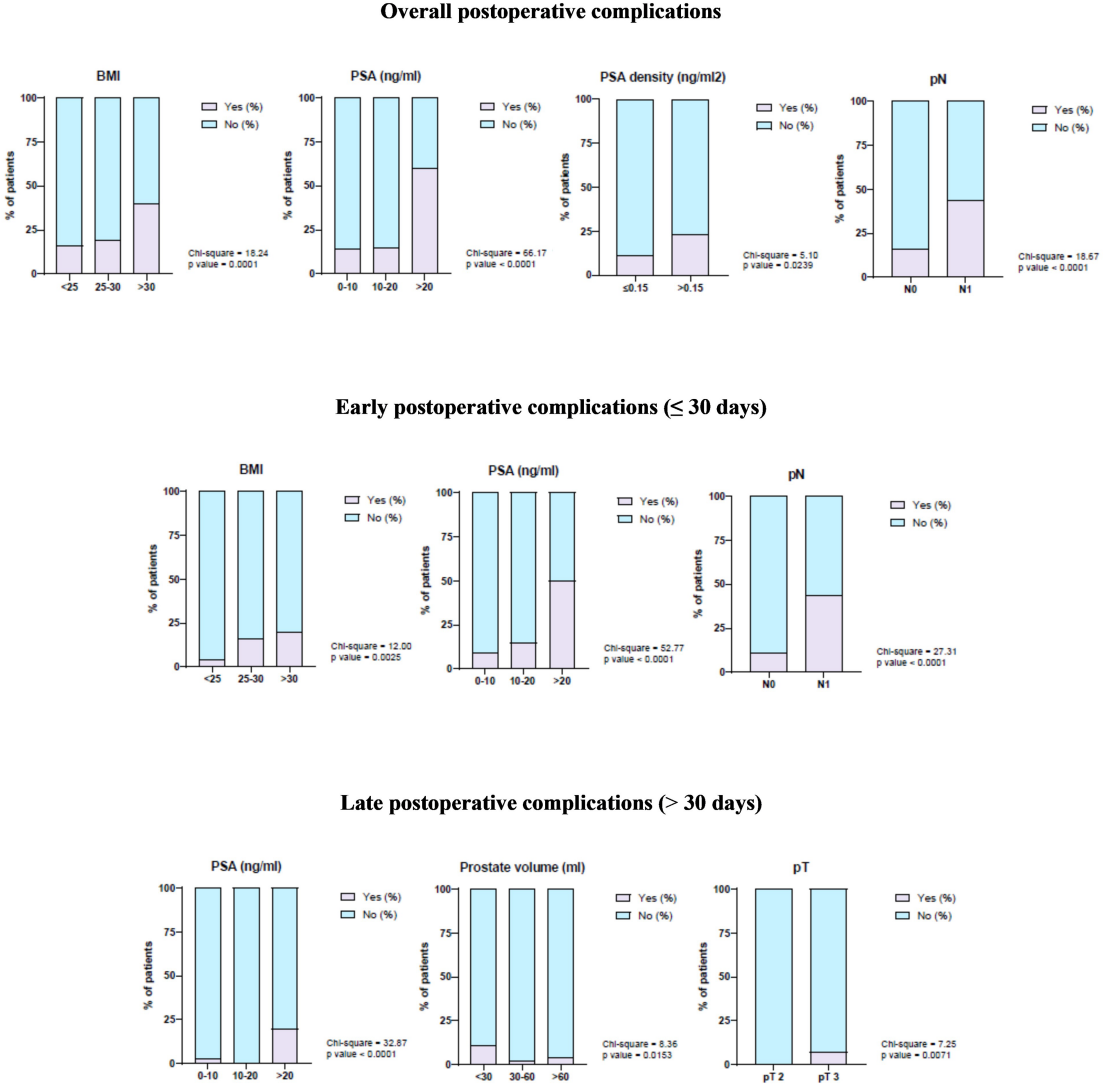
Correlation of demographic and clinical–pathological characteristics with overall postoperative complications, early postoperative complications, and late postoperative complications.

After study population stratification, univariate logistic regression analysis showed that BMI > 30 kg/m^2^, PSA >20 ng/mL, PSA density > 0.15 ng/mL^2^, and pN1 significantly correlated with overall postoperative complications. Moreover, according to the statistical analysis, BMI > 30 kg/m^2^, PSA >20 ng/mL, and pN1 significantly correlated with a higher rate of early complications, while PSA > 20 ng/mL, prostate volume < 30 mL, and pT3 were significantly associated with a higher risk of late complications (Figure 1).

In addition, in multivariate logistic regression analysis, PSA >20 ng/mL significantly correlated with overall postoperative complications, while PSA >20 and pN1 correlated with early complications. It was not possible to perform a multivariate regression analysis of late complications because the numerosity of events was not sufficient (Table 2).

**Table 2:** Multiple logistic regression test.

Overall postoperative complications				
	Ref. value	OR	95% CI	*p*-value
BMI (>30)	<25	2.31	0.14–26.47	0.5161
BMI (25-30)		1.37	0.38–5.95	0.6446
PSA (ng/mL) (10-20)	0–10	0.96	0.21–3.83	0.9539
PSA (ng/mL) (>20)		8.34	1.48–57.56	**0.0205**
Prostate volume (mL) (>60)	<30	3.86	0.44–53.85	0.2552
Prostate volume (mL) (30–60)		5.33	0.87–61.96	0.1126
PSA density (ng/mL^2^) (>0.15)	≤0.15	2.48	0.65–11.59	0.2087
pT (pT3)	pT2	0.48	0.14–1.55	0.2356
pN (N1)	N0	5.14	0.78–33.33	0.0789
Early postoperative complications (≤30 days)
	Ref. value	OR	95% CI	*p*-value
BMI (>30)	<25	13.15	0.43–543.00	0.1301
BMI (25–30)		6.55	0.90–147.90	0.1196
PSA (ng/mL) (10–20)	0–10	1.96	0.36–10.36	0.4220
PSA (ng/mL) (>20)		11.60	1.59–111.90	**0.0207**
Prostate volume (mL) (>60)	<30	5.85	0.31–273.50	0.2910
Prostate volume (mL) (30–60)		10.61	0.83–439.20	0.1312
PSA density (ng/mL^2^) (>0.15)	≤0.15	2.92	0.54–22.66	0.2456
pT (pT3)	pT2	0.27	0.05–1.15	0.0963
pN (N1)	N0	16.05	1.96–168.80	**0.0118**

BMI, body mass index; PSA, prostate-specific antigen.

In bold significant values.

With regard to functional outcomes, our findings showed that at 3, 6, and 12 months, IPSS was of 10.5 ± 4.9, 10.0 ± 5.0, and 10.2 ± 5.3, respectively; urinary continence was recovered in 53 (49.1%), 72 (66.7%), and 86 (79.6%) patients at the same intervals, respectively. With regard to sexual function, preoperative potent patients were 47 in number, and potency recovery occurred in 9 (19.1%), 14 (29.9%), and 17 (36.2%) patients at 3, 6, and 12 months, respectively.

## Discussion

Radical prostatectomy is the treatment of choice for clinically localized PCa in patients with a life expectancy beyond 10 years (1). Despite the fact that PCa screening based on PSA has been associated with a decrease in PCa-related mortality, overdiagnosis, and overtreatment of silent PCa, 20%–30% of patients were diagnosed with high-risk localized and locally advanced PCa (1, 19). According to Gandaglia et al., RARP provides a well-standardized, safe, and oncologically successful treatment choice in highly selected patients with locally advanced PCa (20). RARP with the extraperitoneal approach and PLND for high-risk PCa have a low rate of complications, as well as oncological and functional outcomes comparable to those of tRARP.

However, concerns on the role of eRARP as a treatment for high-risk PCa remain because of a lack of experience of clinicians in some low-volume centers as well as technical difficulties in performing ePLND involving a higher risk of complications.

Intraoperative complications could be related to patient position, trocar insertion, gas insufflation, and surgical technique. The rate of intraoperative complications varies from 0.4% to 1.3%, and the most described in the literature are peripheral and pelvic nerves, ocular, cerebral, thoracic, vascular, bowel, rectal, ureteral, and pressure injuries (21–26).

Di Pierro et al. reported the highest rate (3.4%) of peripheral nerve injuries caused by position (27). In our series, we did not register any case of neurapraxia due to the patient's position. This finding could be explained by the use of a low degree of Trendelenburg position, approximately 15°–18°, and the use of a soft no-sliding pad. The use of the latter associated with a low degree of Trendelenburg position let to avoid shoulder supports use reducing compression on the peripheral nerves. The use of a less steep Trendelenburg position is possible in the extraperitoneal approach, thanks to the natural barrier of the peritoneum on the viscera (10).

Vascular injuries often occur during trocar insertion and lymphadenectomy, but they can also happen when approaching the dorsal vein complex and lateral pedicles or when dissecting the neurovascular bundle (23). Vascular injuries during abdominal access are attributable in most cases to the use of the Veress needle or during first trocar placement. These injuries are rare (0.03%–0.2%), and the vessels most commonly damaged are the aorta and common iliac vessels. These types of lesions occur more frequently when the patient is in the Trendelenburg position during trocar placement, because this position rotates the promontory, bringing the aortic bifurcation closer to the umbilicus (22). However, the most frequent vascular injury involves the inferior epigastric artery during placement of the trocars along the pararectal line. Bleeding from the epigastric artery can be easily identified intraoperatively and can frequently be controlled by bipolar coagulation and suturing. To prevent postoperative bleeding, it is mandatory to carry out an inspection of the surgical cavity with low pressure to verify occult bleeding at the end of the surgery (23).

In this study, we reported the case of one patient with bleeding who needed blood transfusion. The bleeding occurred during pararectal robotic trocar insertion with injury of the inferior epigastric left vein. It was treated intraoperatively by coagulation and clip application. The extraperitoneal approach provides a potential advantage in bleeding management: the Retzius space is a small virtual cavity capable of self-containing and subsequently compressing the vessels and stopping the bleeding itself (10, 11).

Our case series reported a case of very rare intraoperative complication, the pneumothorax. Only a few such cases have been reported in the literature. It is a non-surgical complication that could occur during intubation for bronchus damage or congenital blebs rupture (28). In our case, the pneumothorax was caused by emphysematous blebs rupture and it was treated, without sequelae, by chest drainage after RARP because there was no respiratory impairment.

The most frequent postoperative complications described in the literature are bleeding with pelvic hematoma, urinary leakage, lymphocele, small bowel obstruction, port-site hernia, bladder neck contracture, and thromboembolic complications. Peri- and postoperative complications in RARP have been reported by Novara et al. to range from 3% to 26%, while in a review by Sotelo et al., they have been found to range between 1.9% and 6.8% (23, 29). In a recent meta-analysis by Pucheril et al., the median rate of overall complications was found to be 12.6% but with wide ranges in single studies; in any case, most complications were of minor grade (24). The highest rate of complications has been reported by di Pierro et al., who described complications in 42% of patients (27).

In our case series, overall complications occurred in 17.6% of patients, of which 73.9% were of minor grade and 26.1% of grade III. No grade IV or V were recorded. More specifically, we found 15 early complications and 4 late ones. Thus, our findings are in line with those reported in the literature.

Risk factors reported in the literature for urinary leakage from vesical-urethral anastomosis are obesity, a large prostate, previous prostatic surgery, excessive bleeding, surgeon learning curve, urethral stump length, and integrity of anastomosis during bladder distention (24). The rate of urine leak is reported in 0.1–6.7% of patients, and it could result not only from anastomosis but also from other urinary sites that may have been inadvertently injured during surgery. The incidence of ureteric injury is approximately <1% (30). Most leakages from vesical-urethral anastomosis are diagnosed during the first 10 days after surgery with cystography, CT, or ultrasound. In most cases, the first-line treatment consists in the replacement of the bladder catheter that must be kept in place from a few days up to 3 weeks. Before removal of the bladder catheter, it is recommended to repeat cystography to confirm leakage resolution. If this treatment fails, possibly a surgery can be performed to revise the anastomosis or to place nephrostomies (24).

In recent studies, the placement of pelvic drainage could be safely omitted (31). In our study, two urinary leakages appeared after 72 h from the surgery when bowel function was recovered. We believe that the recovery of intestinal peristalsis could stretch the vesical-urethral anastomosis favoring urinary leakage. For this reason, we consider appropriate to keep a pelvic drain in place at least until bowel motility recovery. Our postoperative management protocol includes the removal of the bladder catheter on the 7th postoperative day together with the pelvic drain. In this way, also late urinary leakage related to bowel recovery may be highlighted by the drain; we also prefer to completely drain small leakages that have the potential to become a single-site infection. We do not perform a routine urethrocystography before the removal of the bladder catheter except in case of pelvic drain production upon a suspicion of urinary leakage from vesical-urethral anastomosis.

In a recent review, Tsaur and Thomas (32) reported the rates of lymphocele to range from 2% to 61%, while Pucheril et al. (24) reported rates of 0.1%– 30.9%. As a risk factor, Capitanio et al. identified a threshold of 20 lymph nodes that were removed, while Naselli et al. demonstrated the performance of an extended template (33, 34). According to the Pasadena consensus panel, in case of an appropriate ePLND, usually ≥10 lymph nodes are retrieved (35). For ePLND, our template included the external and internal iliac vessels and the nodes within the obturator fossa; for the standard PLND, our template included the external iliac vessels and the nodes within the obturator fossa (36). In our case series, the mean number of lymph nodes removed was 12.2 ± 5.3, in line with that of the Pasadena consensus panel. The majority of diagnosed lymphoceles remain asymptomatic. However, a small percentage, approximately 8%–10%, became symptomatic because of enlargement or infection, which may cause voiding dysfunction, lower extremity edema, fever, or, in the worst-case scenario, deep vein thrombosis (32, 37). The surgical approach could impact the rates of lymphocele. tRARP seems to be less associated with lymphocele development because of a reabsorption of lymphatic fluids into the peritoneal cavity. However, Horovitz et al., in their comparison of tRARP and eRARP, reported no significant differences in the rates of lymphocele (9). A meticulous sealing of the lymphatic vessel using clips, thermal energy, and hemostatic agents have been demonstrated to be useful in reducing the risk of lymphocele (32).

Our findings showed an overall lymphocele rate of 10.2% and the symptomatic ones were 1.9%. We always performed a fine sealing of the lymphatics with thermal energy and Hem-o-lok, achieving comparable results to those of major reported case series (38).

Bladder neck contracture following radical prostatectomy represents a late complication, usually occurring in 0.3%–3.2% of patients (24). It is reported in median 5 months after surgery with obstructive and irritative lower urinary tract symptoms (22). These data are comparable to the results of our case series where the two contractions found were successfully treated by endoscopic incision.

Another late complication found in our clinical experience is the anastomotic clip migration, which caused a secondary bladder neck contracture. It was removed by transurethral incision using a Holmium laser. Anastomotic clip migration from prostate lateral pedicles is a rare complication, occurring in <1% of radical prostatectomy patients. The mechanism of surgical clip migration has not been clarified. Kadekawa et al. assumed inflammation around vesico-urethral anastomosis as the main mechanism. Clip migration can lead to bladder neck contracture, obstructive and irritative LUTS, hematuria, urinary infection, and stone formation (21, 39).

The main limitations of this study are its small sample size and the high rate of early dropout.

## Conclusions

RARP with PLND is considered a good first-line therapeutic option, in the setting of multimodal therapy, in patients with high-risk PCa. RARP with the extraperitoneal approach is feasible and safe, and from our experience, we found that it is burdened with only a few intra and postoperative complications, mostly of low grade. In addition, the extraperitoneal approach allows a better management of some complications because bleeding, urinary leakage, as well as lymphoceles, remain circumscribed.

## Data Availability

The raw data supporting the conclusions of this article will be made available by the authors, without undue reservation.
